# Design, construction, and testing of an accurate low-cost humidistat for laboratory-scale applications

**DOI:** 10.1140/epje/s10189-021-00062-5

**Published:** 2021-04-05

**Authors:** Lars B. Veldscholte, Rens J. Horst, Sissi de Beer

**Affiliations:** Sustainable Polymer Chemistry Group, Department of Molecules and Materials MESA+ Institute for Nanotechnology, University of Twente, P.O. Box 217, 7500 AE Enschede, The Netherlands

## Abstract

Stable and precise control of humidity is imperative for a wide variety of experiments. However, commercially available humidistats (devices that maintain a constant humidity) are often prohibitively expensive. Here, we present a simple yet effective humidistat for laboratory-scale applications that can be easily and affordably (<€250) constructed based on an Arduino Uno as microcontroller, a set of proportional miniature solenoid valves, a gas washing bottle, and a humidity sensor. The microcontroller implements a PID controller that regulates the ratio of a dry and humid airflow. The design and implementation of the device, including a custom driver circuit for the solenoids, are described in detail, and the firmware is freely available online. Finally, we demonstrate its proper operation and performance through step response and long-term stability tests, which shows settling times of approx. 30 s and an attainable relative humidity range of 10–95%

**Supplementary Information:**

The online version supplementary material available at 10.1140/epje/s10189-021-00062-5.

## Introduction

Careful control of the humidity in a sample chamber is necessary for numerous experiments ranging from controlling molecular charge transport [1] to designing state-of-the-art sensors [2]. A device that accomplishes this is called a *humidistat* (analogous to a *thermostat* for regulating temperature). In contrast to a thermostat, however, humidity is less easily adjusted; instead of heating the chamber by controlling a heat exchanger or Joule heating element, humid and dry air must be mixed in proper proportions and led into the chamber.

*Relative humidity* is a dimensionless number representing the ratio of the partial pressure of water vapour with respect to its saturation pressure:

1 $$\begin{aligned} \Phi = \frac{p}{p_\mathrm{sat}} . \end{aligned}$$

It is typically expressed as a percentage and ranges from 0 to 100%. When the humidity is pushed beyond 100%, the dew point is reached and water vapour will condense into a liquid. In contrast, *absolute humidity* refers to the mass of water in a volume or mass of air. When not further specified, ‘humidity’ usually refers to relative humidity.

Different inexpensive techniques have been developed to adjust the relative humidity. A commonly employed method is based on thermodynamic equilibria of water vapour with various salt solutions [3–5]. This method is simple and cheap because it does not require any specialised hardware, but is also rather inconvenient for several reasons. First of all, equilibration is often quite slow. More importantly, it does not allow for free choice of values of relative humidity: a particular salt has to be selected for every value of the relative humidity required, which also means that when an experiment covers a range of different humidities, the salt solution has to be swapped out for another every time when moving to another value of humidity, thus interrupting the experiment.

**Fig. 1:**
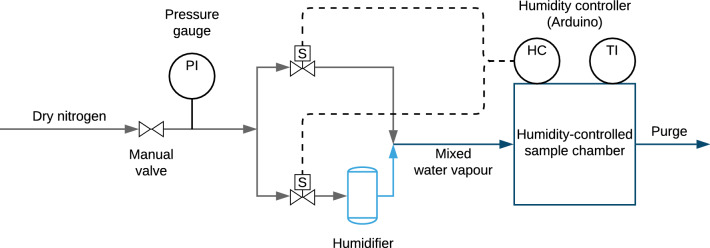
Piping and instrumentation diagram for the humidistat. From left to right: pressure-regulated dry nitrogen from a supply line is split into two. One of the two flows is humidified, while the other is not. After passing through solenoid valves, the flows are combined again in the chamber. Closed-loop control is achieved using a controller measuring the resulting humidity in the chamber and actuating the valves. PI, TI, and HC stand for pressure indicator, temperature indicator, and humidity controller, respectively

Another technique that is commonly utilised is local heating of a water bath in an enclosed chamber [6, 7]. The humid air above the heated bath will diffuse to the sample to be studied. The disadvantage is that this diffusion tends to be slow as well. A faster alternative is to use an air stream consisting of a mixture of dry and water-saturated air guided towards the sample [1, 8–10]. However, the lack of closed-loop control combined with its susceptibility to external disturbances and long response times renders this set-up very arduous to control manually. To overcome these limitations, electro-pneumatic devices (devices involving electronic circuits to control airflows using actuators such as solenoid valves) can be utilised [11].

Major advantages of electro-pneumatic devices are that no collection of chemicals is required, the relative humidity can be freely specified over a broad continuous range, equilibration is fast, and changing to a different value of humidity is as easy as pressing a button on the controller interface. However, the equipment is naturally more complex. Commercial solutions exist, but are often prohibitively expensive and/or not versatile enough because they are tailored specifically to a particular set-up, as noted by others [12–14].

Boulogne [11] describes a design and implementation for a cheap humidistat based on a PID controller. This work is an advancement on that design. Boulogne used simple non-proportional solenoid valves that are either fully ‘on’ or fully ‘off’. Different humidity settings are achieved by varying the on/off times of the solenoids in a method called time-proportional control$^{1}$. This suffices when the chamber is large enough (compared to the flowrates) that its inherent time constant is orders of magnitude larger than the control period. However, when the chamber is much smaller, this fails and one should resort to a more delicate control strategy. Hence, here we use *proportional* solenoid valves controlled by a PWM^1^ signal, which allows analog control over the flowrate through the valves. The choice for proportional solenoid valves incurs a more complex driver. To this end, a driver circuit is designed and built.

## Design and principle

The principle of the humidistat presented in this work is based on mixing a humid (saturated water vapour) and a dry airflow in a chamber. By varying the ratio of the two flows, the full humidity range can be reached. Figure 1 shows the piping and instrumentation diagram of the system. The two flows are regulated by inserting solenoid valves. Humidity control is then realised using a controller measuring the humidity in the chamber and controlling the two valves accordingly, in a closed-loop control scheme.

### PID control

PID (proportional–integral–derivative) control is a common closed-loop control scheme. Its principle is to compare the measured quantity of interest called the *process variable* (PV) to a user-specified *setpoint* (SP). The controller then seeks to close the error between the PV and SP by adjusting the *control variable* (CV), which acts as an input to the plant through the final control element (FCE), e.g. a valve. [15]

Three terms contribute to the CV: the proportional term, which is given by the error between the PV and SP; the integral term, which is given by the integral of the error over time; and finally the derivative term, which is given by the derivative (rate of change) of the error with respect to time. The CV is the sum of each of the three terms multiplied with a gain. This can be expressed by the following equation:

2 $$\begin{aligned} u(t) = K_\mathrm{p}\ e(t) + K_\mathrm{i} \int _0^t e(x)\ \mathrm {d}x + K_\mathrm{d} \frac{\mathrm {d}e(t)}{\mathrm {d}t} \end{aligned}$$

where $K_\mathrm{p}$, $K_\mathrm{i}$, and $K_\mathrm{d}$ are the proportional, integral, and derivative gain, respectively, *u*(*t*) is the control variable, and *e*(*t*) is the error. *x* is a bound variable within the integral. In practice, this continuous PID equation is discretised and the integral and derivative are approximated by finite difference methods.

In this case, the PV is the humidity, as measured using a sensor in the chamber. The user enters the desired SP on the controller interface, and the CV affects the control voltages presented to the solenoid drivers. The two valves are operated counter to each other: one of the valve’s control voltages is reversed. This way, the total flowrate is constant (as long as the valves’ response is linear).

## Implementation

**Fig. 2:**
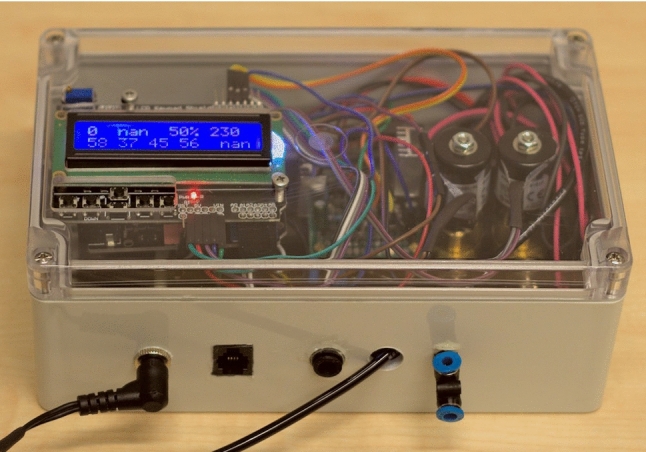
Picture of the finished device, excluding the power supply, gas washing bottles, and chamber. The enclosure contains the Arduino microcontroller (with display and keypad mounted in the lid), solenoid valves, electronics (solenoid drivers, temperature monitor, and wiring between all modules). Dimensions: $200 \times 120 \times 75$ mm

### Hardware

The hardware implementation of the design outlined above consists of the following main components (Fig. 2):

- Microcontroller: Arduino Uno
- Humidity sensor: DHT22
- Proportional miniature solenoid valves (2x): SMC PVQ31
- Solenoid drivers: Custom-built
- Humidifier: Gas washing bottles

An exhaustive bill of materials can be found in sect. 1 of the SI.

#### Microcontroller

For the microcontroller, an Arduino Uno Rev3 is used. The Arduino Uno is one of the most popular boards from the Arduino line of inexpensive open-source microcontroller boards. The Uno is powered by an 8-bit ATmega328P MicroController Unit (MCU) and featuring sufficient flash memory, RAM, and input/output pins for this purpose.

For the user interface, a Keyestudio LCD1602 Expansion Shield is used. This is an expansion board (or ‘shield’) that combines a $16 \times 2$ character monochrome LCD with six push buttons in one package that can conveniently be stacked on top of the Arduino.

#### Humidity sensor

We use a AM2302 (also known as DHT22) for sensing the humidity. This is an simple, small, and inexpensive module that combines a polymer capacitance humidity sensor with a temperature sensor. The manufacturer specifies a 0.1% resolution, and a typical accuracy of 2%, diverging at the two extrema to 5% [16]. Independent testing revealed these specifications to be reliable, barring some long-term drift, and non-negligible temperature-dependent error [5].

The specified sampling rate is only ${0.5}{\hbox { Hz}}$, but we found we could poll it four times as quickly (at ${2}{\hbox { Hz}}$) without any apparent adverse effects. The increased polling rate allows for a correspondingly shorter timestep of the PID control loop. Smith [17] found that ‘overclocking’ the sensor like this may skew the results due to self-heating of the sensor, but we did not find any evidence for this in our set-up at ${2}{\hbox { Hz}}$, likely because the appreciable airflow in the chamber provides sufficient cooling.

**Fig. 3:**
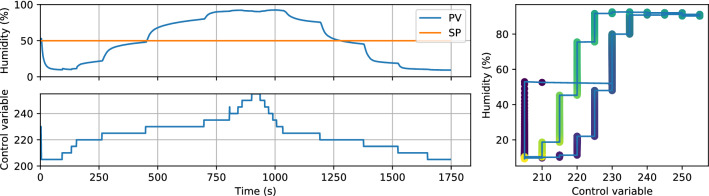
PV response to open-loop test in when the CV is increased in steps of 10. The PV is allowed to stabilise before moving to the next value of CV

#### Solenoid valves

For regulating the flow of humid and dry air, two solenoid valves are used. Solenoid valves employ a solenoid that electromagnetically actuates the plunger of a valve. *Proportional* solenoid valves are solenoid valves that provide analog control over the flowrate by allowing the valve to be partially opened, in contrast to ordinary (non-proportional) solenoid valves that are either fully open or fully closed.

By using two proportional solenoid valves (one in the dry airflow, and the other in the humid airflow), the ratio of dry to humid airflow can be continuously varied, while the total flowrate can be kept constant.

We use two PVQ30-series miniature proportional solenoid valves from SMC. These valves are normally closed direct-acting valves with a hysteresis of less than 10%. We selected the PVQ31-6G-16 model, which has an orifice diameter of ${1.6}{\hbox { mm}}$ which allows for at most ${7}{\hbox { bar}}$ operating pressure and a flowrate of at most ${100}\hbox { L }\hbox {min}^{-1}$ and uses the ${12}\hbox { V}$ version of the solenoid. [18]

#### Solenoid drivers

The solenoid valves cannot be powered by the Arduino directly, because the Arduino cannot supply the voltage and current required. Therefore, a simple electronic circuit is designed and built that drives the solenoid according to a control signal from the Arduino.

The solenoids can be driven by a simple variable direct current, but in practice, PWM is more commonly used. This has multiple reasons: first of all, the Arduino Uno used does not have any true DC output capability, so an external DAC (digital-to-analog converter) would be required. Second, to some degree driving a solenoid valve by PWM or a smoothed PWM current is beneficial for reducing hysteresis in the valve because the ripple in the current helps to overcome the stiction (static friction) that lies at the basis of this hysteresis.

The flowrate through a solenoid valve is controlled by the current flowing through the solenoid. The SMC PVQ31-6G-16 solenoid valve used requires ${330}\hbox { mA}$ at full flow [18]. For driving the solenoids, a VCCS (voltage-controlled current sink) driver circuit is designed and built. The VCCSs power the solenoids, which are controlled by signals from the Arduino. We refer to sect. 2 of the SI for a detailed description.

### Humidifier

Water vapour for humid air is generated by passing air through gas washing bottles (DURAN laboratory bottle with Drechsel-type head with filter disc). Two bottles are connected in series to ensure sufficient water vapour saturation at higher flowrates.

### Firmware

The firmware for the MCU was written in C++ with the Arduino Core and the PlatformIO build system for AVR-GCC. It consists of classes with the following tasks:

- A controller class which ‘wraps’ a class implementing a PID loop
- User interface using the buttons and LCD
- Communication over serial (USB) with a Python script for real-time monitoring and logging
- Temperature monitoring (optional)

The PID class is heavily inspired by the Arduino PID library by Beauregard [19].

The humidistat can be used in fully standalone manner with the integrated buttons and displayed as a user interface. When monitoring and/or recording of the parameters (sensor values, setpoint, PID terms, temperatures) is desired, it is possible to connect to a PC over USB. A Python script for this purpose is included.

The source is available online under a free license [20].

**Fig. 4:**
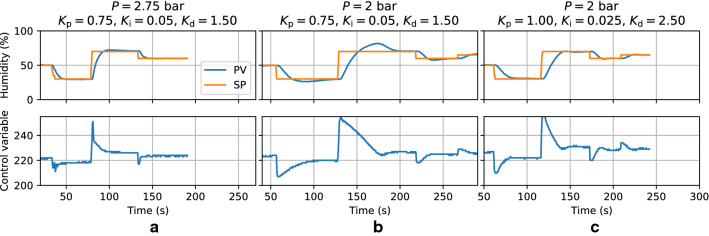
Response of the system to step changes for a properly tuned high flowrate (${1}{-}{4}\hbox { L }\hbox {s}^{-1}$) case (**a**), an improperly tuned low flowrate ($<{1}\hbox { L }\hbox {s}^{-1}$) case (**b**), and finally a properly tuned low flowrate case (**c**). **a** and **b** correspond to tuning #2, while **c** corresponds to tuning #1. The setpoint is manually stepped from 50%, to 30%, 70%, 60%, and finally 65%. The PV is allowed to stabilise to within 1 point of the setpoint

## Test results and discussion

**Fig. 5:**
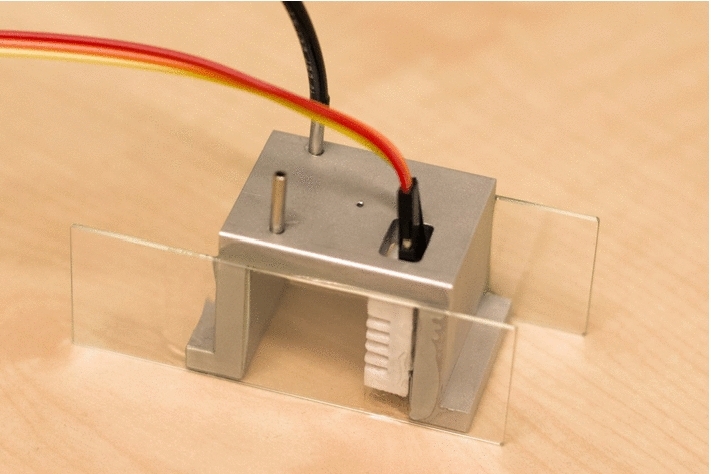
Picture of the chamber. This chamber is intended for contact angle measurements. Inside dimensions: $3 \times 3 \times 2.5$ $\hbox { cm}$

The humidistat was subjected to testing with a chamber (Fig. 5) designed to fit a small (max $3 \times 3 \hbox { cm}$) sample for contact angle measurements. The total inside volume of the chamber is ${22.5}\hbox { mL}$. Flowrates used are on the order of magnitude of ${1}\hbox { L }\hbox {min}^{-1}$, which results in typical residence times on the order of seconds. All measurements are conducted at room temperature ($\sim {21}\,^{\circ }{\mathrm{C}}$).

### Tuning of controller parameters

The control scheme employed requires tuning of the proportional, integral, and derivative gains for proper operation. In this case, this is complicated by the nonlinearities exhibited by the plant. First of all, the flowrate through a solenoid valve is not exactly linear with the coil current. In our set-up, where two solenoid valves are operated counter to each other and the two flows are combined, this also means that the total flowrate does not add up to a perfectly constant value. This has implications for the dynamics of the system: for instance, the system’s response time is for a large part composed of the residence time of air in the chamber which is dependent on the flowrate. The solenoid valves also exhibit quite severe hysteresis arising from plunger stiction [21, 22]. This is visible in sect. 3 of the SI, where the flowrate through a valve is plotted versus the duty cycle. The effect these valve properties have on the integrated plant can be seen in the open-loop test depicted in Fig. 3: the same step of 10 in CV does not increase the PV in a linear manner. Hysteresis is also clearly visible in the PV versus CV plot. Hence, standard PID tuning methods such as Ziegler–Nichols [23] fail.

In addition to the three PID gains, two additional parameters are subject to adjustment: the interval (timestep) of the control loop and the minimum duty cycle. For the former, lower is usually better because it reduces dead time and improves stability, but it is limited by the temporal resolution of the sensor; if the timestep of the control loop is shorter than the temporal resolution of the sensor, the controller only ‘sees’ sporadic (large) changes in the PV which leads to undesirable behaviour. We use an interval of ${500}\hbox { ms}$, in line with the maximum sampling rate of the humidity sensor. The minimum duty cycle arises from the fact that the solenoid valves have a (rather large) deadband (DB): no air is permitted through the valves until about ${265}\hbox { mA}$ (or about 80% of the full-flow current) is achieved. The exact value depends on the inlet pressure [18]. Hence, the usable PWM range is only from this minimum value to 100%. This value has to be measured and set in the controller firmware. When it is incorrectly set, it impairs the controller performance: when it is set too low, deadband remains at the low side of the valves. When it is set too high, the valves cannot be fully closed. The minimum duty cycle was simply determined by identifying the highest value at which no flow is permitted.

### Dynamic performance

Despite the challenges in PID tuning resulting from imperfect actuators, reasonably satisfactory manual tunings were achieved (Table 1).

**Table 1:** Tuning parameters

#	P (bar)	$K_\mathrm{p}$	$K_\mathrm{i}$ ($\hbox {s}^{-1}$)	$K_\mathrm{d}$ (${\hbox {s}}$)	DB
1	2	1.00	0.025	2.50	210
2	2.75	0.75	0.050	1.50	205

Corresponding responses to step changes are shown in Fig. 4. Good controller performance is characterised by the PV tracking the setpoint (SP) as closely as possible: by moving to and stabilising on new values quickly (command tracking), and by deviating from the setpoint as little as possible under external influences (disturbance rejection). The controller is sensitive to the inlet pressure (and thereby, to the resulting total flowrate), and the optimal controller parameters depend on this. For example, at high flowrates a higher $K_\mathrm{i}$ (integral gain) helps to reduce settling times for large setpoint step changes, but at lower flowrates the plant’s inherent response time and dead time are substantially longer, causing the integral term to build up excessively, leading to overshoot and ultimately hurting settling times [24]. This is visible in Fig. 4: the more ‘aggressive’ tuning #2 is suitable for high flowrates (${1}{-}{4}\hbox { L }\hbox {s}^{-1}$, 4a), but when applied with a lower flowrate ($<{1}\hbox { L }\hbox {s}^{-1}$), it leads to overshoot (Fig. 4b). The more conservative tuning #1 in this case offers better performance, stability, and robustness at varying pressures (Fig. 4c).

Settling times depend on the setpoint step size, but are at most on the order of ${30}{\hbox { s}}$ (for the humidity to stabilise to within 1 point of the setpoint) for the low flowrate test case.

### Range

The humidistat is able to achieve humidity values ranging from 10 to 95% at room temperature. The limiting factors for the extrema are the dryness of the feed air, efficacy of the humidifier, and air-tightness of the chamber. As we use a dry laboratory nitrogen supply as feed, this is not a limiting factor for the minimum humidity. However, in case that the air feed is not fully dry, this obviously imposes a limit on the lowest humidity achievable. When very low humidities are still desired, a desiccator can be added to ensure adequate dryness.

Furthermore, we found that the chamber has to be adequately sealed in order to achieve extreme humidity values. When the chamber (which is open on the bottom, Fig. 5) was not placed on a flat surface, too much mixing with ambient air occurs and it becomes difficult to achieve extreme humidities. Naturally, this also depends on the residence time (and hence, the flowrate) of air in the chamber. This leads to a non-trivial dependence of the maximum humidity on the flowrate: if the chamber is too open to the ambient atmosphere, increasing the flowrate improves the maximum achievable humidity. However, excessively high flowrates can also decrease the maximum achievable humidity when the flowrate through the gas washing bottles is too high to allow for complete saturation. When this is the case, one observes the paradoxical effect of a lower resulting humidity for the highest CV values. This is slightly visible in Fig. 3 as a inverse slope in CV–PV curve at the end of the CV range.

### Accuracy and precision

The accuracy and precision of the humidistat are foremost limited by the sensor, which are 2% and 0.1%, respectively. However, the precision in humidity is additionally limited by the quantisation error arising from the digital PWM resolution of the control variable, which is reduced by the relatively high deadband; when a deadband value of 210 is used, this only leaves $255 - 210 = 45$ discrete values to be assumed by the control variable. Intuitively, this implies that a 0.1% precision cannot be reached stably (assuming a self-regulating process). Yet, in practice the humidistat is still able to maintain a humidity with only very minimal variations by occasionally oscillating the control variable between two values: in essence an intrinsic form of dithering. An example of this is visible in sect. 4 of the SI, where a long-term (${1}{\hbox { h}}$) stability test demonstrates the PV stays rather constant barring some $<0.2$ point oscillations as the control variable switches between two adjacent values.

## Conclusion and outlook

We presented the design and implementation of a DIY humidistat aimed at laboratory-scale applications that allows for reliable, fast regulation of humidity over a wide range (10–95%). The device was tested with a small chamber with a volume of ${22.5}\hbox { mL}$, and at flowrates on the order of magnitude of ${1}\hbox { L }\hbox {min}^{-1}$. Step response testing shows that the humidity stabilises to within 1 per cent point of a new value within ${30}{\hbox { s}}$. The humidity stays constant in the chamber at long timescales and does not drift. The firmware source and solenoid driver design are published alongside.

Some improvements to this design are conceivable. An effective technique to improve performance and stability of the controller is to utilise cascaded PID control, where the humidity controller does not directly actuate the valves, but instead a cascade of control loops is set up, where the outer loop which controls the humidity regulates the setpoints of the inner loops. The inner loops control the flow through each valve using mass flow sensors. This essentially constitutes a mass flow controller. The benefits of such a control scheme stem from the fact that the inner flow control loop is much faster: its PV (the flowrate) can respond much more rapidly than the outer PV (the humidity in the chamber), yielding better dynamic performance. However, this would add some complexity and considerable cost.

Another option that does not require more costly hardware is to employ a more sophisticated control scheme. Techniques exist for correcting for actuator imperfections such as valve stiction by augmenting the PID scheme with a stiction model-based compensator [22]. Although this approach adds considerable additional complexity, it might be worth to consider if stiction proves overly problematic.

A less-radical improvement would be to modify the voltage divider/RC-filter stage in the solenoid driver to not only scale the maximum voltage, but also shift the lowest voltage so it corresponds to the deadband in the solenoid valves, permitting usage of the full PWM range and thereby increasing CV resolution. This simple modification to the solenoid driver circuit would reduce the CV quantisation error, which leads to further reduction in the slight steady-state PV oscillations shown in sect. 4 of the SI.

It is possible to reduce the number of components required by swapping the set of 2-port solenoid valves for a single 3-port (directional) solenoid valve, simplifying the design. An additional benefit of this choice is that the nonlinear response is less problematic, because the total flowrate is always a linear combination of the two inlets. However, we were unable to source suitable 3-port proportional solenoid valves.

Some improvement to accuracy and precision may be attainable by upgrading to a more accurate sensor, like the slightly more expensive Sensirion SHT71. This sensor offers tighter specifications and better reliability [25]. It is also slightly smaller.

Finally, the $16 \times 2$ character LCD proved rather spartan for this application. A larger, higher-resolution, graphical LCD would be an attractive upgrade, though at the cost of more complex interfacing.

## Supplementary Information

Below is the link to the electronic supplementary material.

Supplementary material 1 (pdf 1356 KB)

## Footnotes

1 PWM refers to pulse width modulation. Note that time-proportional control can be regarded as equivalent to PWM, except on much longer timescales.
