# Supplementary material for: Design, construction, and testing of an accurate low-cost humidistat for laboratory-scale applications
Source: Eur Phys J E Soft Matter. 2021 Apr 5;44(4):48. doi: 10.1140/epje/s10189-021-00062-5 (PMC8021525; doi:10.1140/epje/s10189-021-00062-5)
Supplement: Supplementary file 1 — Supplementary material 1 (pdf 1356 KB) [file 10189_2021_62_MOESM1_ESM.pdf]

Design, Construction, and Testing of an Accurate Low-cost Humidistat for Lab-scale Applications: Supplementary information

Lars B. Veldscholte, Rens J. Horst, and Sissi de Beer

Sustainable Polymer Chemistry Group, Department of Molecules & Materials
MESA+ Institute for Nanotechnology, University of Twente
P.O. Box 217, 7500 AE Enschede, the Netherlands

2021-02-15

1 Bill of Materials

In table 1, an as comprehensive as possible bill of materials is given. Some items are not listed individually, such as electric wiring and connectors and pneumatic tubing and couplings, because they are typically ordered in bulk and only small quantities are needed for the construction of this device.

Table 1: *Bill of materials with (rounded) prices (per unit, where applicable).*

Component	Product	Price (€)
**Electronics**		
Microcontroller	Arduino Uno (compatible)	7.00
Display and buttons	Keyestudio LCD1602 Expansion Shield	6.00
Power supply	5A 12V DC power supply	9.00
Humidity sensor	DHT22	4.50
**Solenoid driver**		
Perfboard	4x6 cm perfboard	0.70
Dual opamp	LM358	0.80
MOSFET (2x)	IRLZ34N	1.20
MOSFET heatsink (2x)	TO-220 Cu heatsink	0.80
Capacitor (2x)	10 µF 50 V electrolytic capacitor	0.10
Flyback diode (2x)	1N4007	0.02
Various resistors	3x 3 Ω, 330 Ω, 1 kΩ, 4.7 kΩ	0.50
Misc. wires, headers, connectors		
**Temperature monitor** (optional)		
Perfboard	4x6 cm perfboard	0.70
Thermistor (4x)	MF5A-3 10K NTC	0.25
Resistor (4x)	10 kΩ	
Misc. wires, headers, connectors		
**Pneumatics**		
Solenoid valves (2x)	SMC PVQ31	80.00
Gas washing bottle (2x)	DURAN laboratory bottle with Drechsel-type head with filter disk	
Tubing	Festo PUN-H 3,4,6 mm OD	
Couplings	Festo Push-in coupling QSM mini	
**Finishing**		
Enclosure	True components TC-9065484	25.00
Power jack	True components TC-9065484	4.00
6P4C jack	Encitech 2101-0100-14	1.00

2 Electronic design of solenoid driver

2.1 Motivation: simple voltage source solenoid driver

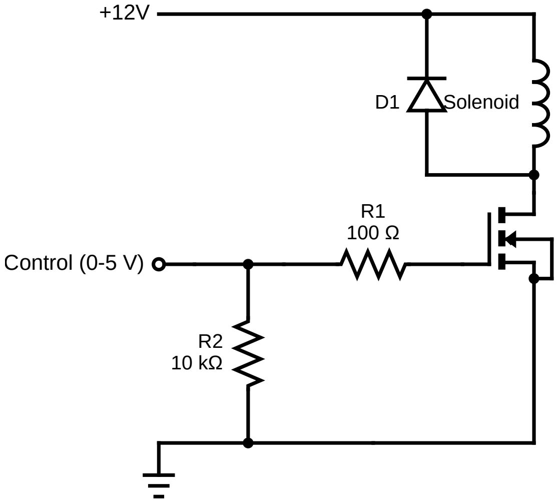

Figure 1: *Circuit diagram for the simple (constant-voltage) solenoid driver using a MOSFET.*

A simple solenoid driver consisting of a MOSFET switching the solenoid powered by a 12 V supply, with the PWM signal from the Arduino controlling the MOSFET gate (fig. 1) was initially tried but deemed inadequate. The reason is that such a circuit constitutes a constant-voltage source: for some control signal, a corresponding voltage is supplied to the load.[1] However, the quantity of interest is the solenoid's *current*, as this corresponds to the flowrate, and the solenoid's resistance changes rather considerably when it warms up. With the simple MOSFET driver this presented a significant problem for the repeatability of the system as it would not respond equally when cold compared to when warmed up.

2.2 Design

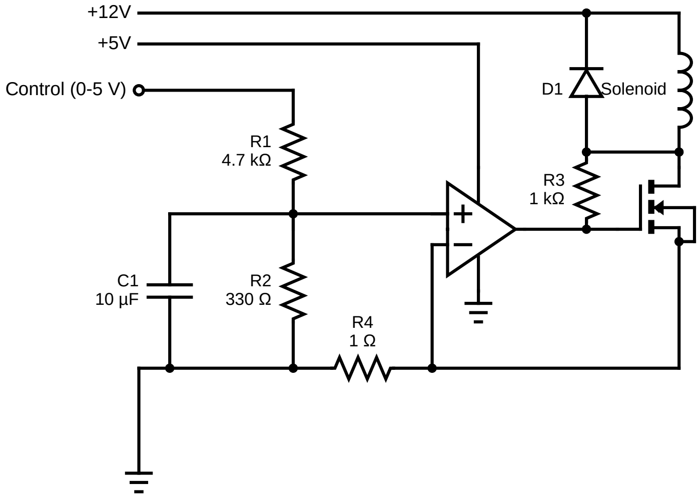

Figure 2: *Circuit diagram for the solenoid driver employing a voltage divider and filtering stage and a voltage-controlled current sink (VCCS).*

[1] More accurately, the PWM signal presented at the MOSFET's gate is also presented to the load: i.e. it is switched between 12 V and 0 V, with the duty cycle being equal to that of the control signal. Because the solenoid is an inductor, the resulting current will be heavily flattened; the solenoid inherently smooths the PWM signal.

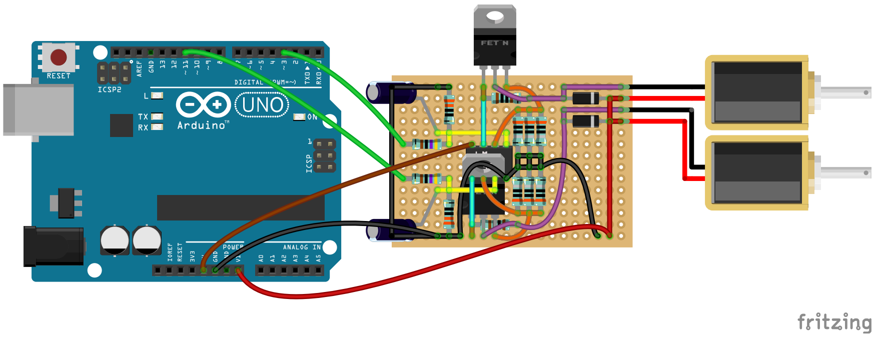

Figure 3: *Perfboard layout of the VCCS (2x) connected to the Arduino Uno and the solenoids.*

To alleviate this problem, a more sophisticated driver was designed (fig. 2). This new driver consists of a OPAMP (OPerational AMPlifier) that forms a voltage-controlled current source (or more accurately *sink*, since it is used as a low-side switch) by driving the MOSFET gate according to the difference between the non-inverting input $V_{\text{in}+}$, connected to the Arduino's control signal, and the inverting input $V_{\text{in}-}$, connected to a $1\,\Omega$ current sense resistor (R4). This resistor is placed between the MOSFET source and ground, and is used to measure the load current (as the voltage drop corresponds directly to it). The opamp amplifies the difference between the two inputs $V_{\text{in}+} - V_{\text{in}-}$. Since IN- is connected to the current sense resistor, this provides negative feedback. The result is that the opamp aims to conform the load current to the signal on $V_{\text{in}+}$ by driving the MOSFET gate V_{g} appropriately. As such, it constitutes a VCCS with a transconductance of $1\,\text{A}\,\text{V}^{-1}$ (since the current sense resistor has a value of $1\,\Omega$): putting a voltage of $1\,\text{V}$ on $V_{\text{in}+}$ will cause $1\,\text{A}$ of current to flow through the load, provided the supply voltage is sufficient. [1]

The resistors R1 and R2 form a voltage divider, which purpose is to scale the $0\,\text{V}$ to $5\,\text{V}$ control signal from the Arduino to the desired voltage range of $0\,\text{V}$ to $0.33\,\text{V}$ (corresponding to the maximum solenoid current of $330\,\text{mA}$).

The VCCS does not work as intended with the raw PWM signal from the Arduino, because it would cause the opamp to simply saturate at all times: it will try to conform the solenoid current to the PWM signal presented at $V_{\text{in}+}$, but it will never be able to do that because the solenoid's inductance limits the rate of change of current. Hence, it is necessary to filter the PWM signal *before* the VCCS. This is accomplished by adding a capacitor C1 between IN+ and ground. This forms a RC filter which transforms the rectangular PWM wave into a smoothened triangular wave with a smaller amplitude (but with ample remaining ripple) that the solenoid current is able to follow. This diversion from a pure PWM signal does however incur some switching losses from the MOSFET, causing it to dissipate some power [2]. To prevent overheating, a small heatsink is added onto the MOSFET.

The MOSFET is biased using a $1\,\text{k}\Omega$ drain-to-gate resistor (R3) to introduce negative feedback and thereby stabilise the circuit. Without it, the MOSFET switching the highly inductive load combined with parasitic capacitances caused ringing [3], which turned the entire circuit unstable. A common alternative way of stabilising a VCCS is to utilise a compensator in the form of a capacitive coupling between the output and the inverting input of the opamp [4].

2.3 Implementation

A 1N4007 diode is connected anti-parallel (reverse-biased) to the solenoid as a freewheeling diode to eliminate inductive flyback that would otherwise exceed the MOSFET's breakdown voltage and overload it in the process.

For the switching device, a IRLZ34N logic-level n-channel enhancement-mode power MOSFET is used. For the opamp the LM358 is used, which contains two independent opamps in one package. This is convenient because two solenoid drivers are required, which can be built using a single LM358.

2.4 Analysis

The performance of the circuit was evaluated using the circuit simulator software Qucs-S [5], allowing us to simulate the transient response of the circuit. The results were confirmed empirically by measuring V_{R4} and V_{g} on the completed device using a oscilloscope.

The VCCS circuit (fig. 2) was modelled in Qucs-S using models of the actual components (LM358 opamp and

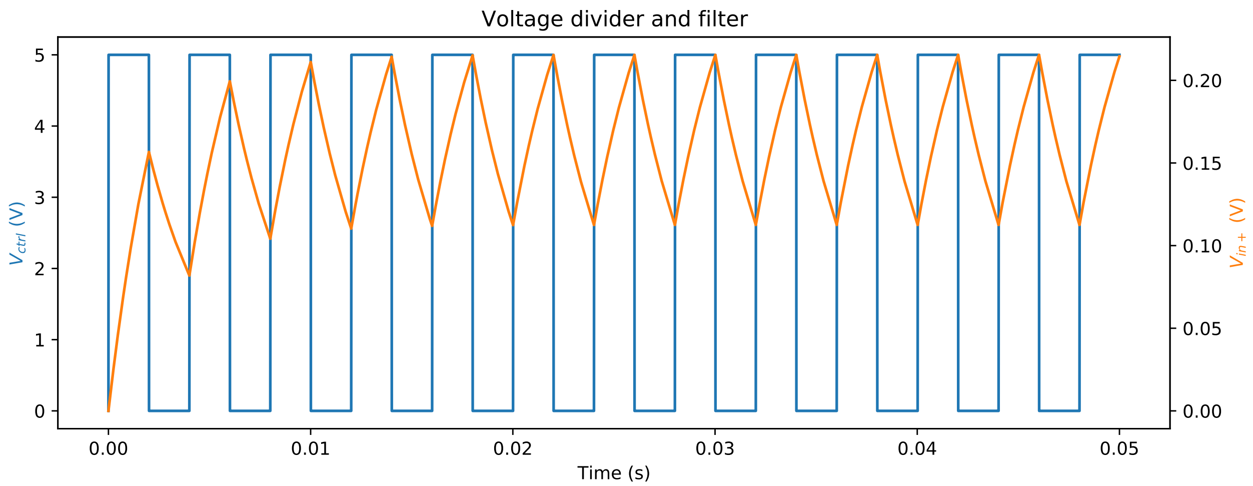

Figure 4: *The voltage divider and filter capacitor transforms the* 0 V *to* 5 V *rectangular PWM wave into a smoothened* 0 V *to* 0.33 V *wave with a smaller amplitude. Shown is a 50% duty cycle* 250 Hz *control signal.*

IRLZ34N MOSFET). The load (the solenoid) was modelled as a resistor in series with a inductor. The resistance was taken as 36 Ω and the inductance was estimated as 10 mH. [2]

2.4.1 Compliance

The compliance of the circuit is determined by two factors: the drive voltage V_{drive}, and the load inductance L_{l}.

Already in the DC case, the drive voltage V_{dr} together with the load resistance R_{l} imposes an upper bound on the current:

$$I_{\max} = \frac{V_{\text{dr}}}{R_{\text{l}}} \tag{1}$$

However, when considering the time-dependent behaviour, the load inductance also plays a role. The inductance limits how quickly the current can change. Specifically, the load current will not be able to follow the requested current if the L/R time constant is longer than the RC time constant of the filtering stage.

$$\frac{L_{\text{l}}}{R_{\text{l}}} < R_{\text{f}} C_{\text{f}} \tag{2}$$

Filling in the values for our circuit, this means that with $V_{\text{dr}} = 12\,\text{V}$, R_{l} should be less than $\frac{V_{\text{dr}}}{R_{\text{l}}} = \frac{12}{0.328} \approx 36.59\,\Omega$ to achieve full current ($I_{\text{l}} = 0.328\,\text{A}$). Additionally, at that value of load resistance, the load inductance cannot exceed $\tau_{\text{RC}}\ R_{\text{l}} = \frac{1}{1/4700+1/330}\ 10 \cdot 10^{-6} \cdot 36.59 \approx 0.11\,\text{H}$.

[2] The solenoid's inductance was not specified by the manufacturer and is non-trivial to estimate from first principles due to unknown coil specifications and its moving core. Still, simulation results of the circuit with $R_{\text{l}} = 10\,\text{mH}$ agree reasonably well with empirical observations of the completed device.

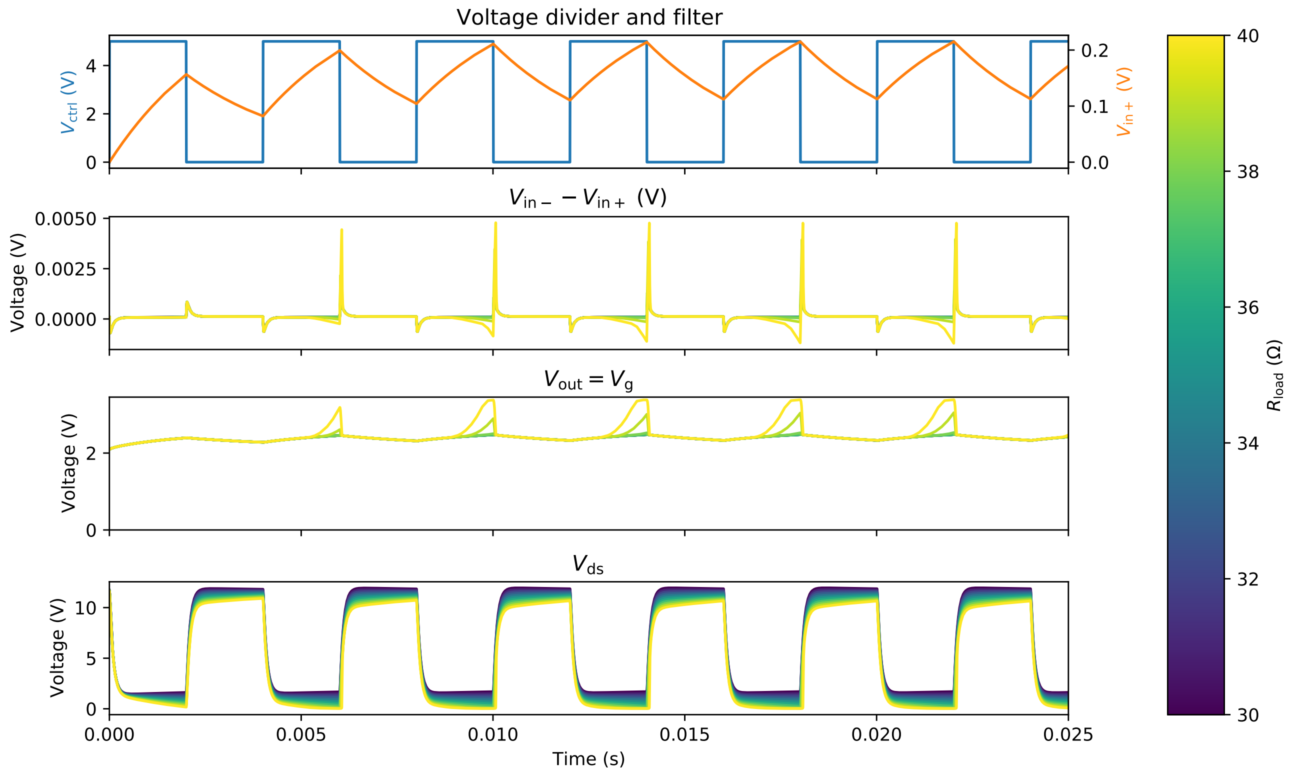

Figure 5: *Transient response of the VCCS with 50% duty cycle* $250\,\mathrm{Hz}$ *control signal. The load resistance is swept between* $30\,\Omega$ *to* $40\,\Omega$*, while its inductance is fixed at* $100\,\mathrm{mH}$.

3 Solenoid valve flow characterisation

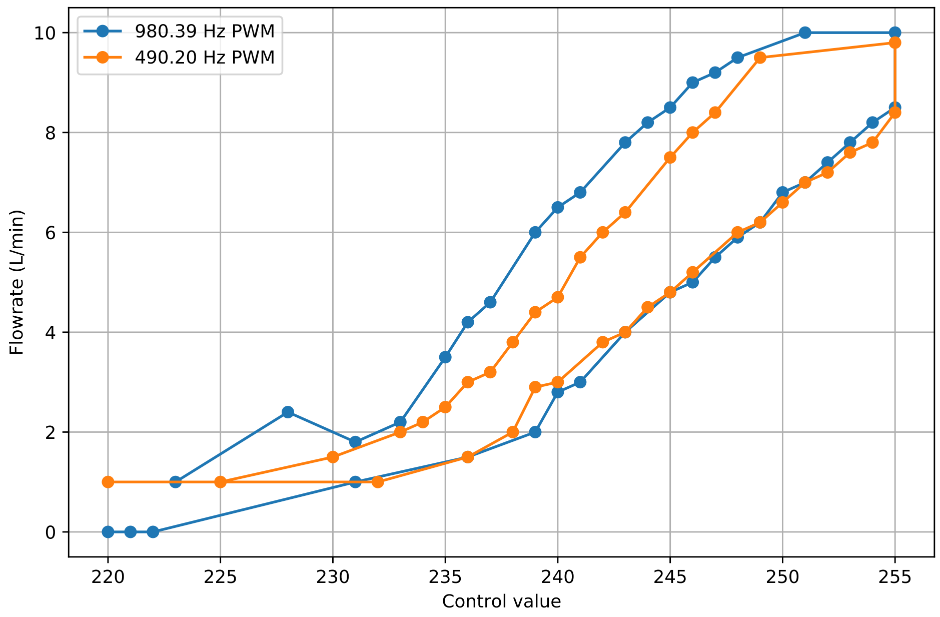

Figure 6: *Characterisation of the solenoid valve's hysteresis by measuring the flowrate as a function of the control value (duty cycle) for two values of the PWM frequency.*

4 Long-term stability test

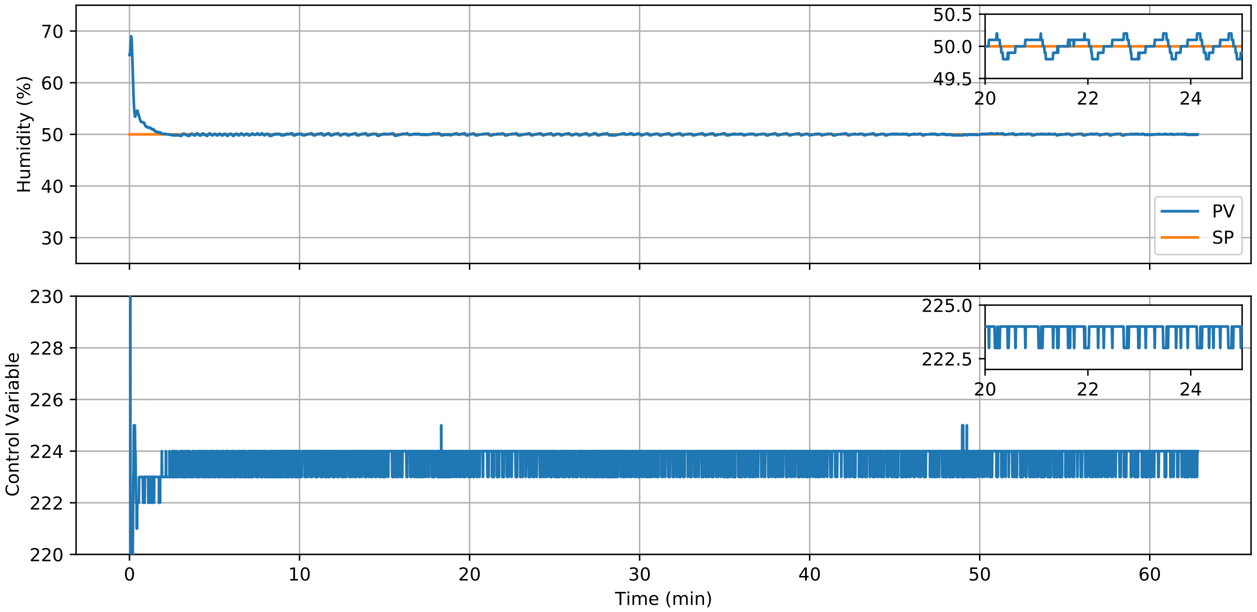

Figure 7: *Long-term response of the system with a constant setpoint of 50%. Note the slight oscillations in the PV arising from quantisation error in the CV, which is switching between two adjacent values.*

References

[1] P. Horowitz and W. Hill. *The Art of Electronics*. Cambridge University Press, 2015. ISBN: 978-0-521-80926-9.

[2] I. Batarseh. "4 - The Power MOSFET". In: *Power Electronics Handbook (Third Edition)*. Ed. by M. H. Rashid. Boston: Butterworth-Heinemann, 2011, pp. 43–71. ISBN: 978-0-12-382036-5. DOI: 10.1016/B978-0-12-382036-5.00004-5.

[3] Toshiba. *Parasitic Oscillation and Ringing of Power MOSFETs*. 2018. URL: https://toshiba.semicon-storage.com/info/docget.jsp?did=59456 (visited on 2021-02-10).

[4] G. Satterfield. *Programmable low-side current sink circuit*. 2018. URL: https://www.ti.com/lit/pdf/SLAA868 (visited on 2021-02-10).

[5] M. Brinson and V. Kuznetsov. "Qucs-0.0.19S: A new open-source circuit simulator and its application for hardware design". In: 2016 International Siberian Conference on Control and Communications (SIBCON). 2016, pp. 1–5. DOI: 10.1109/SIBCON.2016.7491696.
